# Replication of *TCF4* through Association and Linkage Studies in Late-Onset Fuchs Endothelial Corneal Dystrophy

**DOI:** 10.1371/journal.pone.0018044

**Published:** 2011-04-20

**Authors:** Yi-Ju Li, Mollie A. Minear, Jacqueline Rimmler, Bei Zhao, Elmer Balajonda, Michael A. Hauser, R. Rand Allingham, Allen O. Eghrari, S. Amer Riazuddin, Nicholas Katsanis, John D. Gottsch, Simon G. Gregory, Gordon K. Klintworth, Natalie A. Afshari

**Affiliations:** 1 Department of Biostatistics and Bioinformatics, Duke University Medical Center, Durham, North Carolina, United States of America; 2 Center for Human Genetics, Duke University Medical Center, Durham, North Carolina, United States of America; 3 Duke University Eye Center, Duke University Medical Center, Durham, North Carolina, United States of America; 4 Wilmer Eye Institute, The Johns Hopkins University School of Medicine, Baltimore, Maryland, United States of America; 5 Center for Human Disease Modeling, Duke University, Durham, North Carolina, United States of America; 6 Department of Pathology, Duke University Medical Center, Durham, North Carolina, United States of America; Innsbruck Medical University, Austria

## Abstract

Fuchs endothelial corneal dystrophy (FECD) is a common, late-onset disorder of the corneal endothelium. Although progress has been made in understanding the genetic basis of FECD by studying large families in which the phenotype is transmitted in an autosomal dominant fashion, a recently reported genome-wide association study identified common alleles at a locus on chromosome 18 near *TCF4* which confer susceptibility to FECD. Here, we report the findings of our independent validation study for *TCF4* using the largest FECD dataset to date (450 FECD cases and 340 normal controls). Logistic regression with sex as a covariate was performed for three genetic models: dominant (DOM), additive (ADD), and recessive (REC). We found significant association with rs613872, the target marker reported by Baratz *et al*.(2010), for all three genetic models (DOM: *P* = 9.33×10^−35^; ADD: *P* = 7.48×10^−30^; REC: *P* = 5.27×10^−6^). To strengthen the association study, we also conducted a genome-wide linkage scan on 64 multiplex families, composed primarily of affected sibling pairs (ASPs), using both parametric and non-parametric two-point and multipoint analyses. The most significant linkage region localizes to chromosome 18 from 69.94cM to 85.29cM, with a peak multipoint HLOD = 2.5 at rs1145315 (75.58cM) under the DOM model, mapping 1.5 Mb proximal to rs613872. In summary, our study presents evidence to support the role of the intronic *TCF4* single nucleotide polymorphism rs613872 in late-onset FECD through both association and linkage studies.

## Introduction

Fuchs endothelial corneal dystrophy (FECD), first described by Ernst Fuchs [1], is a common progressive disorder of the corneal endothelium that typically becomes symptomatic during the fifth or sixth decade of life [2], [3], although corneal endothelial abnormalities can be clinically detected several years before patients become symptomatic [4]. The disorder affects as much as 4% of the United States population over the age of 40 years [5]–[7] and occurs predominantly in women, who comprise approximately 75% of cases [8]. This debilitating disorder leads to corneal edema with a loss of corneal clarity, painful episodes of recurrent corneal erosions, severe impairment of visual acuity, and sometimes even blindness.

FECD is often inherited as an autosomal dominant trait [5], [9]–[13] and 50% of clinical cases of FECD are estimated to show familial clustering [2]. While it is recognized that FECD has a genetic basis, few genes have been identified that explain the genetic underpinnings of FECD susceptibility. The first gene to be causally linked with FECD was *COL8A2* (MIM: 120252) on chromosome 1, in which two missense mutations, p.L450W and p.Q455V/Q455K, have been replicated in rare early-onset (before 40 years of age) FECD multigenerational families [3], [13]–[15] and in atypical sporadic FECD cases [15]. Additional *COL8A2* missense mutations have been identified in late-onset FECD patients, although their role remains unclear due to conflicting results [16]–[18]. Mutations in two additional genes that play roles in other corneal endothelial dystrophies have also been suggested to cause FECD. *SLC4A11* (MIM: 610206), which causes autosomal recessive congenital hereditary endothelial dystrophy (CHED2, MIM: 217700) [19], was found to contain mutations in Chinese, Indian, and Caucasian patients with the common late-onset phenotype of FECD [20], [21]. Additionally, mutations in *TCF8* (MIM: 609141), whose loss of function causes posterior polymorphous corneal dystrophy (PPCD, MIM: 609141) [22], were recently reported in late-onset Caucasian [23] but not in Chinese FECD patients [24].

Several genome-wide linkage scans using large multigenerational families have reported FECD loci on chromosomes 13 (*FCD1*), 18 (*FCD2*), 5 (*FCD3*), and 9 (*FCD4*) [23], [25]–[27]. Our own genome-wide linkage scan using affected sibpairs (ASPs) from 21 late-onset FECD families and one large multigenerational family [28] detected five regions of linkage with multipoint LOD scores >1.5, including the chromosome 1p region near *COL8A2*. Neither our late-onset FECD families nor our large, multigenerational family replicated the four previous FECD loci (*FCD1* to *FCD4*), but the number of families used in the study may have been underpowered to detect significant linkage.

The first genome-wide association study (GWAS) for FECD was reported recently, using an initial dataset of 130 unrelated cases and 260 unaffected controls genotyped with the Illumina 370K BeadChip panel [29]. After genotyping their most significant findings in a replication dataset containing 150 cases and 150 controls, Baratz and colleagues concluded that a single nucleotide polymorphism (SNP) on chromosome 18q21, rs613872, in an intron of a gene encoding transcription factor 4 (*TCF4*, MIM: 602272) showed genome-wide significant association with FECD (*P* = 1.10x10^−12^ for the initial GWAS dataset; *P* = 1.79x10^−13^ for the replication dataset; *P* = 2.34x10^−26^ for the combined dataset).

Here we present additional evidence for the presence of a FECD locus on chromosome 18. We report the results from an expansion of our previous linkage study, as well as an association study analyzing the association of rs613872 with FECD in a dataset containing 450 unrelated FECD cases and 340 unaffected controls, the largest sample of FECD patients interrogated to date.

## Materials and Methods

### Ethics Statement

Our study was performed in accordance with the Declaration of Helsinki and the institutional review boards at Duke University Medical Center and Johns Hopkins University (JHU) specifically approved this study. Both FECD study sites, the Cornea Clinics at Duke University Eye Center (DUEC) and the Wilmer Eye Institute at JHU, obtained the appropriate institutional review board approval for research on human subjects prior to initiating subject recruitment, and all individuals gave written, informed consent. The control subjects from the Duke glaucoma genetics study were also recruited under the approval of the Duke Institutional Review Board, and consented to allow their biological samples to be used by other research studies.

### Subjects and Families

All FECD subjects underwent detailed ophthalmic examination, including slit lamp biomicroscopy, to determine FECD severity. Grading of disease severity was determined using a slightly modified version of the Krachmer scale classification system [2], which classifies severity on a scale of 0 to 5: (1) grade 0: no central cornea guttae; (2) grade 1: scattered central cornea guttae; (3) grade 2: 1 or 2 mm of central cornea guttae; (4) grade 3: 2 to 5 mm of grouped cornea guttae; (5) grade 4: >5 mm of grouped central cornea guttae; (6) grade 5: cornea guttae with corneal edema. Subjects were classified as affected if grade ≥2, unaffected if the corneal examination was normal (grade  = 0), and unknown if grade 1.

Two Caucasian datasets were used for association and linkage studies. The association dataset consisted of 450 unrelated FECD cases, each with a Krachmer grade ≥2, and 340 unaffected controls with age at enrollment of ≥45 years old. The 450 unrelated cases used here were either probands from the family dataset (see below) or singleton cases, all of whom were ascertained through the cornea clinic at DUEC. The unrelated controls were from 26 unaffected married-in spouses from the DUEC family dataset and 314 control subjects from the glaucoma genetic study at DUEC [30]. Glaucoma control subjects underwent detailed eye examination and had no signs of corneal abnormalities at the time of subject enrollment.

The linkage dataset consisted of 64 multiplex families (at least two affected individuals per family) containing a total of 215 subjects (69.8% females). Families were ascertained independently from the Cornea Clinics at DUEC or at the Wilmer Eye Institute at JHU. Demographic data, including age at enrollment and gender, for the individuals analyzed in the association and linkage datasets are summarized in **Table 1**.

**Table 1:** Demographic data for individuals analyzed in the linkage and association datasets.

Dataset	Variable	Summary
Linkage	No. of families (DUEC, JHU)^a^	64 (40, 24)
	No. of samples (No. of affected)	215 (165)
	No. of ASPs^b^	81
	Male, Female	65, 150
	Mean (SD^c^) of age^d^ in years	62.47 (13.6)
Association	No. of samples (affected, unaffected)	790 (450, 340)
	Male, Female	280, 510
	Mean (SD) of age in years	66.8 (10.9)

a DUEC: Duke University Eye Center; JHU, Johns Hopkins University.

b ASPs: affected sibpairs (ASP) count is based on n-1 for n siblings per family^c^SD: standard deviation.

d Age: age at the time of study enrollment.

### Marker Selection and Genotyping

Association analysis focused on the SNP rs613872 in *TFC4*, the most significantly associated SNP in the GWAS performed by Baratz and colleagues [29]. We also genotyped rs10490775 in *PTPRG* on chromosome 3, which was significant but not at a genome-wide level in the Baratz GWAS, to examine possible replication in our dataset. The two SNPs were genotyped with pre-designed TaqMan® allelic discrimination assays (Life Technologies, formerly Applied Biosystems, Inc., Foster City, CA), which use unlabeled polymerase chain reaction (PCR) primers and two allele-specific probes containing the TaqMan® minor groove binding group (MGB) probe and either a FAM™ and VIC® dye label in a 384-well plate format. PCR reactions were performed with Taqman® Universal PCR Master Mix on the GeneAmp® PCR System 9700 (Applied Biosystems, Inc.), and the ABI7900HT Fast PCR System (Applied Biosystems, Inc.) was used for reading allelic discrimination calls. Quality control (QC) samples, including two CEPH (Centre d'Etude du Polymorphisme Humain) pedigree individuals, one no-template sample, and two duplicate samples (one male, one female), were contained within each quadrant of each 384-well plate. These QC samples were used to provide duplicate samples within one quadrant, across quadrants within one plate, and across plates. Results of the CEPH and QC sample genotyping were compared to identify possible sample plating errors and genotype-calling inconsistencies, and none were observed. A threshold of 95% genotyping efficiency was required for submission to the analysis database.

Two Illumina (San Diego, CA) SNP linkage panels were used for genome-wide linkage marker genotyping. Seventy-two of the 215 linkage samples were included in our previous linkage study [28], and were genotyped using the Illumina GoldenGate linkage panel IVB, which contains 5,858 SNPs. The remaining 143 samples were genotyped using the Illumina Infinium HumanLinkage-12 platform containing 6,090 SNPs, of which 4,811 overlap with the GoldenGate IVB platform. All genomic DNA samples were prepared at a concentration of 75 ng/µL, in a total volume of 10 µL, and were genotyped in four multiplexed assays according to the manufacturer's protocols. All DNA samples were extracted from blood using the PureGene system (Gentra Systems, Minneapolis, MN).

### Data cleaning for the association dataset

Reproducibility of genotypes was examined for the replicate QC samples located in each 384-well Taqman plate to assess genotyping quality. Reproducibility rates of 100% were observed. The two SNP markers, rs613872 and rs10490775, were examined for possible deviations from Hardy-Weinberg equilibrium (HWE).

### Data cleaning for the linkage dataset

We instituted several QC measures to determine the final set of markers for analysis. We first used the Illumina BeadStudio program to check genotype reproducibility rates using the replicated samples, family relationships, gender status of each DNA sample using Y chromosome markers, sample genotype call rates >99%, and the GenCall score which is a quality measure for each genotype used in the Illumina genotyping system. In particular, the GenCall score measures how close a genotype is to the center of the cluster of other samples assigned the same genotype, as compared with the centers of the clusters of the other genotypes, and ranges from 0 to 1. The higher the GenCall score the more reliable the genotype. A set of clean markers was chosen based on GenCall scores ≥0.15, as well as meeting the other QC measures described previously, for further data cleaning.

For additional data quality assurance, 1000 markers with approximately equal inter-marker distances across the genome were selected to examine family relationships using the RELPAIR [31] and PREST [32] programs. After correcting for family relationship errors, Mendelian inheritance inconsistencies were checked using the PEDCHECK program [33]. Missing genotypes were assigned to the family for those markers with Mendelian inheritance inconsistencies.

All SNPs were tested for deviations from HWE. We randomly chose one affected individual per family to include in an unrelated affected dataset, and one unaffected individual per family was selected to include in an unrelated, unaffected dataset. An exact test implemented in the Genetic Data Analysis (GDA) program was used to test HWE, by performing 3,200 permutations to estimate the empirical *p*-value for each marker [34]. We applied a Bonferroni correction (significance threshold = 0.05/total number of markers) to determine significant deviation from HWE. Markers that were out of HWE in the unaffected dataset were excluded in the linkage analysis.

### Association Analysis

Genotypes of rs613872 (T and G nucleotide polymorphism) were tested using three genetic models: dominant (DOM), additive (ADD), and recessive (REC). The genotypes of (GG, TG, and TT) were coded as (1, 1, 0) for DOM; (2, 1, 0) for ADD; and (1, 0, 0) for REC where allele G is the rare allele. A logistic regression association analysis in PLINK [35] was applied with gender as a covariate. We also stratified our association dataset by gender and tested for association of this marker within the males and females. A nominal significance level of 0.05 was applied to declare significant association. The relationship between disease severity (grade level) and genotypes of rs613872 was examined using Fisher Exact test, because some genotype-phenotype has small number of samples, particularly for grade 4.

### Linkage analysis

Two-point parametric linkage analyses using the FASTLINK [36] and HOMOG [37] programs (http://linkage.rockefeller.edu/, provided in the public domain by Rockefeller University, New York, NY) were performed to generate heterogeneity logarithm of the odds (HLOD) scores. Since the mode of inheritance is unknown for FECD, both DOM and REC genetic models were assumed in the parametric analysis with disease allele frequencies of 0.001 and 0.01 respectively. The penetrance (chance of an individual being affected if carrying the disease susceptibility genotype) was based on the assumed genetic models, and was consistent with the goal of conducting an affecteds-only parametric linkage analysis.

MERLIN [38] was used to perform both multipoint parametric and non-parametric linkage analyses (NPL). The same DOM and REC models described above were assumed in the MERLIN parametric analysis. It is known that linkage disequilibrium (LD) may inflate the type I error of a multipoint linkage analysis, particularly when parental genotype data are not available—as is the case for most of our families [39]. We set a threshold of the squared Pearson correlation coefficient (r^2^) between markers at 0.16 in MERLIN to ensure independence between markers for multipoint linkage analysis.

## Results

### Association results

We tested rs613872 in our case-control association dataset of 450 cases and 340 controls; **Table 1** summarizes the demographic data of our subjects. We did not observe deviation from HWE in the control group for rs613872 (*P* = 0.60), indicating high quality genotyping results. However, we found strong deviation from HWE for this marker in the FECD case group (*P*<0.001). This may be the source of the highly significant association signal we observed in our dataset for all three genetic models (DOM: *P* = 9.33×10^−35^; ADD: *P* = 7.48×10^−30^; REC: *P* = 5.27×10^−6^). The effect size of the minor (risk) allele (G) in our dataset was strong: (1) DOM: odds ratio (OR) = 8.01, confidence interval (CI) of OR = (5.80; 11.29); (2) ADD model: OR = 5.35, CI =  (4.00; 7.14); (3) REC: OR = 4.06, CI =  (2.22; 7.42). These results are consistent with the findings of Baratz and colleagues [29]. **Table 2** shows the comparison of genotype frequencies between our FECD cases and controls to the genotype frequencies observed in the FECD GWAS cases and controls studied by Baratz and colleagues. Clearly, the FECD case group has excessive heterozygous genotypes for rs613872 compared to the controls in both datasets, which is consistent to the observation of deviation from HWE in FECD cases. As for rs10490775 in *PTPRG* on chromosome 3, we did not detect significant association with FECD (DOM *P* = 0.98; ADD *P* = 0.96; REC *P* = 0.92).

**Table 2:** Comparison of genotype frequencies between Duke dataset and dataset used in Baratz et al. (2010), and between male and female in Duke dataset for rs613872.

	Duke dataset		Baratz et al.		Duke dataset Male		Duke dataset Female	
	Case	Control	Case	Control	Case	Control	Case	Control
Genotype	N* = 450	N = 340	N = 280	N = 410	N = 123	N = 153	N = 322	N = 182
**GG**	0.15	0.04	0.1	0.02	0.18	0.03	0.14	0.05
**GT**	**0.66** **	0.3	**0.61**	0.25	**0.72**	0.27	**0.63**	0.33
**TT**	0.2	0.66	0.29	0.73	0.10	0.70	0.23	0.62

*N: total sample size.

**Heterozygous frequencies are elevated in all datasets and are highlighted in bold.

When we examined gender-specific groups, we observed the same elevation of the heterozygous (GT) genotype in cases relative to controls in both genders (Table 2). The SNP rs613872 is still significant in both genders (male *P* = 3×10^−26^; female *P* = 1.2×10^−16^). However, this result may not reflect the true level of association due to the small sample size, particularly for males. Furthermore, the results between genders should not be compared due to the unbalanced sample sizes. Among our 450 cases, we have 235 cases with grading information. We did not find the correlation between disease severity and genotypes of rs613872 (P = 0.13). However, more samples may be needed to make a solid conclusion.

### Linkage scan samples and markers

After implementing QC measures for the linkage dataset we removed five individuals from three families due to relationship errors that could not be resolved and five individuals due to low sample call rates (<95%). In total, we analyzed 215 individuals in 64 families (**Table 1**), including 41 ASPs from 64 families, making this the largest dataset used in a genome-wide linkage scan for FECD to date. We noted a high proportion of females in both our linkage and association datasets, likely because of the known gender imbalance among FECD patients [8].

A total of 7,291 markers were genotyped between the two SNP genotyping platforms, with 4,551 markers overlapping between the two chips. Of those, 4,533 markers met our QC criteria for linkage analyses, which included having a GenCall score ≥0.15, a genotype call frequency ≥95%, and no significant deviation from HWE. Eleven markers produced Mendelian inheritance inconsistencies within three families and were assigned missing genotypes within those families. In the multipoint analysis using MERLIN, 3,927 markers met our LD criteria and were analyzed.

### Linkage regions

Eighteen SNPs on ten chromosomes gave two-point HLOD scores ≥2 in either the DOM or REC models (**Table 3**). In particular, three markers produced HLOD scores above 3: rs1889974 (chromosome 10, 119.34 cM, HLOD = 3.37) and rs235512 (chromosome 15, 61.47 cM, HLOD = 3.53) under the DOM model and rs893186 (chromosome 19, 110.25 cM, HLOD = 3.56) under the REC model. Among all markers listed in **Table 3**, only rs998876 on chromosome 5 (194.22 cM) overlaps with a previously identified FECD linkage locus, *FCD3* [27]. **Table 4** summarizes the eight linkage regions that produced peak multipoint LOD scores ≥1.5 in any of the three multipoint linkage analyses, the nonparametric (NPL) and parametric (DOM and REC) analyses. The boundaries of a linkage region outlined in **Table 4** are based on the chromosomal interval that covers one LOD score below the peak marker's LOD score. Two separate regions of linkage were found on chromosomes 16 (28.27–36.15 cM and 56.82–96.27 cM) and X (59.18–83.83 cM and 98.35–124.34 cM), implying that multiple genes on the same chromosome could be influencing FECD.

**Table 3:** Markers with HLOD >2 in either dominant (DOM) or recessive (REC) model from the FASTLINK/HOMOG two-point linkage analysis.

Chromosome	SNP marker	deCODE map (cM)	DOM HLOD score	REC HLOD score
1	rs726344	52.86	1.57	**2.20**
1	rs491603	56.95	1.64	**2.54**
5	rs476569	64.08	1.64	**2.02**
5	rs1301475	78.25	**2.22**	0.61
5	rs998876	194.22	**2.00**	1.59
8	rs2466216	40.75	**2.65**	0.51
8	rs9797	46.84	**2.06**	1.37
8	rs1380229	88.89	**2.26**	0.41
9	rs1407392	113.18	1.14	**2.62**
9	rs1923433	113.20	1.86	**2.34**
10	rs1889974	119.34	**3.37**	1.83
15	rs235512	61.47	**3.53**	1.84
18	rs4941043	78.82	1.99	**3.00**
19	rs7937	66.65	0.80	**2.19**
19	rs893186	110.25	1.30	**3.56**
20	rs674630	30.36	**2.17**	1.41
X	rs1207480	73.97	1.46	**2.70**
X	rs1990383	166.56	**2.08**	0.07

Abbreviation: SNP, single nucleotide polymorphism; cM, centiMorgans; HLOD, heterogeneity logarithm of the odds.

**Table 4:** Results of the multipoint linkage analysis using MERLIN.

Chr	Peak and boundary SNPs	deCODE map (cM)	NPL (LOD score)	DOM (HLOD score)	REC (HLOD score)
6	rs1563512	120.96	0.64	0.63	0.00
	rs988693 (peak)	127.33	1.29	**1.74**	0.19
	rs1894641	129.27	1.07	1.36	0.28
9	rs4077800	92.50	0.58	0.68	0.37
	rs1819730 (peak)	109.99	**1.55**	1.04	1.16
	rs1405	122.92	0.71	0.70	0.02
16	rs887864	28.27	0.25	0.08	1.04
	rs734826 (peak)	31.98	0.84	0.39	**2.29**
	rs2384933	36.15	0.73	0.31	1.42
16	rs13143	56.82	0.59	0.14	0.01
	rs149156 (peak)	84.70	**1.60**	1.08	0.19
	rs1922604	95.27	0.59	0.41	0.65
18	rs1873191	69.94	0.80	1.05	0.00
	rs1145315 (peak)	75.58	1.48	**2.50**	0.08
	rs955427	85.29	0.43	1.50	0.00
19	rs1058511	105.07	0.01	0.00	0.71
	rs1542039 (peak)	108.49	0.12	0.00	**1.71**
	rs893179	109.90	0.08	0.00	1.51
X	rs2024917	59.18	0.87	0.00	0.68
	rs1155699 (peak)	67.14	**1.56**	0.25	1.37
	rs979605 (peak)	67.42	**1.56**	0.25	1.36
	rs1327476	83.83	0.88	0.23	0.05
X	rs897918	98.35	0.69	0.24	0.17
	rs1558022 (peak)	115.73	**1.70**	0.56	0.11
	rs9856	124.34	0.73	0.85	0.20

Note: All linkage regions with a LOD score >1.5 at the peak marker in at least one of the multipoint analyses, nonparametric (NPL), dominant (DOM), or recessive (REC), are presented. The upper and lower bounds of each linkage peak interval presented were selected based on covering all markers with a LOD or HLOD score within one LOD score unit of the peak marker's LOD score. The peak LOD scores are indicated with **bold text**. Chr, chromosome; SNP, single nucleotide polymorphism; LOD, logarithm of the odds; HLOD, heterogeneity logarithm of the odds.

**Figure 1:**
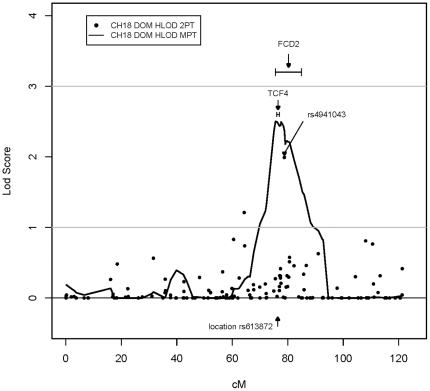
Plot of the top multipoint linkage peak on chromosome 18. SNP markers are plotted along the x-axis by their deCODE map position, and the LOD/HLOD scores for each marker are plotted along the y-axis. The results of the FASTLINK/HOMOG dominant two-point analysis are indicated with black circles, and the results of the MERLIN dominant multipoint analysis are indicated with a black line. The SNP rs4941043 is the peak marker from the two-point analysis (see **Table 3**). The location of the *FCD2* peak (Sundin *et al.*, 2006) and the most significantly associated SNP, rs613872, from the FECD GWAS performed by Baratz *et al.* (2010) are indicated by arrows. The location of the *TCF4* gene is also indicated for reference. 2PT, two-point results; MPT, multipoint results; cM, centiMorgans; LOD, logarithm of the odds; HLOD, heterogeneity logarithm of the odds.

The most promising multipoint linkage region was on chromosome 18 from 69.94 cM to 85.29 cM with a peak multipoint HLOD = 2.5 at rs1145315 (75.58 cM) under the DOM model. The nonparametric analysis showed consistent results for this region with a peak multipoint HLOD = 1.48 at the same peak marker (**Table 4**). In addition, rs4941043 within this same interval produced a HLOD score = 1.99 under the two-point DOM model (**Table 3**). This multipoint linkage peak on chromosome 18 overlaps with the *FCD2* peak reported by Sundin *et al.* [26] and with the most significant GWAS hit, rs613872 in *TCF4*, reported by Baratz *et al.* [29] (**Figure 1**). The SNP rs613872 in *TCF4* is only 1.4 Mb away from our peak DOM multipoint marker, rs1145315.

## Discussion

To date, progress towards identifying the genetic underpinnings of FECD has been limited to a handful of genes, including *COL8A2*, *SLC4A11*, and *TCF8* [3], [13], [20], [21], [23] that have arisen from candidate gene or genome-wide linkage studies. Linkage scans have additionally identified four genetic loci (*FCD1* to *FCD4*) that appear to influence familial FECD [23], [25]–[27]. In spite of these insights, knowledge of the genetic basis of non-familial FECD has remained limited. Recently, Baratz and colleagues identified significant statistical association between a SNP in *TCF4* and FECD in the first genome-wide association study carried out for FECD [29]. Their study identified an intronic SNP in *TCF4*, rs613872, with highly significant allelic and genotypic *p*-values of 2.34×10^−26^ and 1.29×10^−18^, respectively, in their combined analysis of their small discovery and replication datasets.

Here, we present a large case-control association dataset that replicates these recent genome-wide association data [29], revealing a highly significant association between rs613872 and FECD with a *p*-value of 9.33×10^−35^ (DOM). Additionally, we show that the best multipoint linkage region from our family dataset is also located on chromosome 18 (68.94 cM to 85.29 cM), with a peak multipoint HLOD = 2.5 at rs1145315, 1.5 Mb away from rs613872 in *TCF4*. The consistent findings in both our linkage and association studies for an association of rs613872 with FECD together with the findings of Baratz *et al*. suggest that the association is probably not spurious but, rather, is due to certain recombination events in this region that may increase susceptibility to FECD. Additionally, the excess of heterozygous genotypes at rs613872 in both our cases and those of Baratz *et al.* warrants further investigation. Although we replicated the association of rs613872 with FECD in our dataset, we failed to replicate the association with rs10490775 in *PTPRG*. Since we also did not detect evidence of linkage to chromosome 3, we hypothesize that either this locus does not influence FECD risk or its effect on FECD risk is small.

It is of great interest to know if rs613872 genotypes can predict disease severity. Our 235 cases with grading information did not show significant correlation to the genotypes of rs613872, which is consistent to the finding from Riazuddin et al. [40], where 180 cases were used. Although both studies obtained the same observation, the limited sample sizes are the drawback for making a solid conclusion. Many of our early samples were given disease status diagnosis rather than detailed grading information. As our recruitment of FECD cases moving forward, we will be able to increase the number of cases with grading. The disease severity vs. genotypes of any target marker will be able to be evaluated properly.

**Figure 2:**
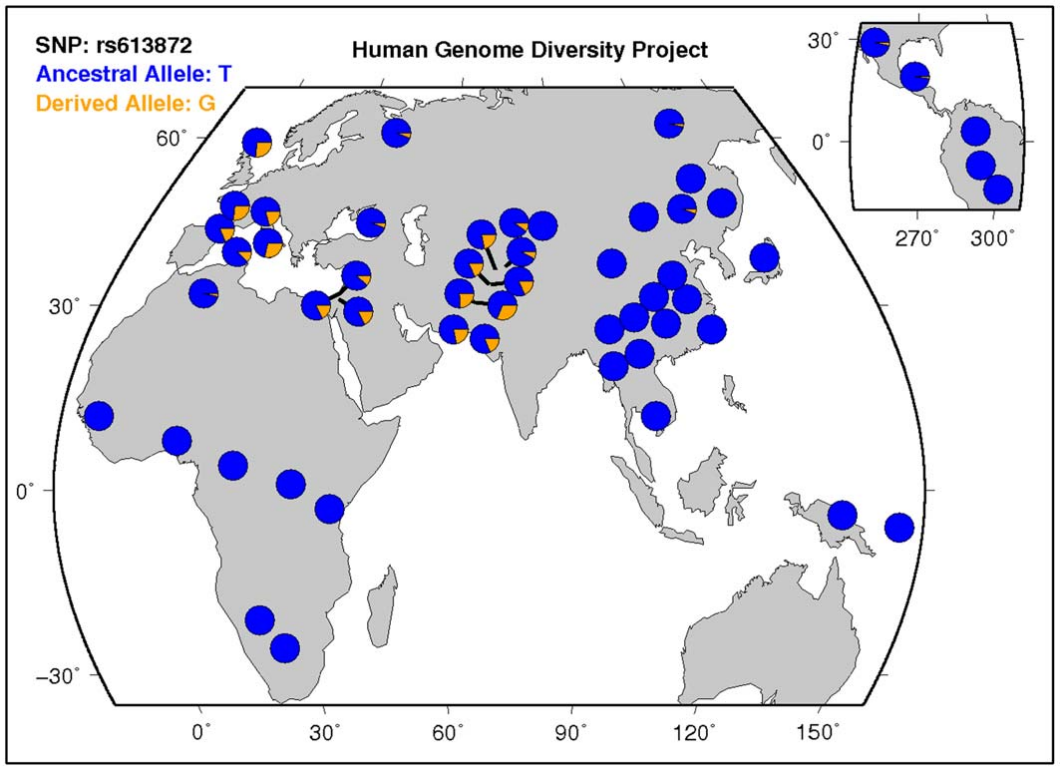
Worldwide distribution of the minor (risk) allele, G, of rs613872 in *TCF4.* Data from the Human Genome Diversity Project, available online through the UCSC genome browser at http://genome.ucsc.edu/. Note the higher prevalence of the risk allele in sample populations from Europe, the Middle East, and Southern Asia and the absence of the risk allele in sample populations from Africa, Eastern Asia, and Central and South America.

According to data from the Human Genome Diversity Project, the minor (risk) allele, G, of rs613872 is rare to nonexistent in populations from Africa, Eastern Asia, and Central and South America, yet is more frequent in European, Middle Eastern, and Southern Asian populations (**Figure 2**, [41]). Given that the FECD cases analyzed by our group and by Baratz *et al*. are of European descent, it will be important to examine whether the association between rs613872 and FECD risk replicates in other ethnic and racial populations. It is possible that alternative *TCF4* risk alleles may be associated in other ethnic and racial populations, which would argue for this locus being functionally important in FECD pathogenesis. Alternatively, if the true FECD disease variant is in LD with rs613872, a lack of association in other ethnic and racial populations may help explain why the disorder has a lower prevalence in other populations like African, East Asian, and South American populations.

GWAS studies commonly raise the question of how to interpret the biological significance of statistically associated SNPs, which may be located in intronic or intergenic regions with no obvious connection to the disease phenotype. Baratz and colleagues did not find any coding variations within the *TCF4* gene that might help explain the functional mechanism behind the association of rs613872 with FECD, so clearly further studies are needed to detect such variations if they exist. Although *TCF4* is an attractive candidate gene within the *FCD2* locus, much remains unknown. If *TCF4* plays a role in familial FECD, further research is needed todiscover the mutation(s) within *TCF4* that segregate with disease in families that show evidence of linkage to *FCD2*. l. Until such a causative mutation(s) is identified, restraint needs to be exercised in not drawing premature conclusions that a causal link between the TCF4 protein and FECD has been identified.

Expression data have indicated that *TCF4* is expressed in eye tissues, particularly in the corneal endothelium [29], [42]–[46], However, *Tcf4* mutant mice do not appear to contain vision/eye abnormalities as a phenotypic feature [47], [48]. Additionally, ENCODE data on the UCSC genome browser [49] (March 2006 assembly) shows that rs613872 is contained within the chromatin immunoprecipitation sequence (ChIP-seq)-purported binding site for two transcription factors, *Ini1* (*SMARCB1*) and *Brg1* (*SMARCA4*). These are both components of the SWI/SNF chromatin remodeling complex that is required for transcriptional regulation of genes normally repressed by chromatin [50], [51]. Given that *TCF4* is a transcription factor, it is possible that variation at this site might have an effect on the spatiotemporal expression of *TCF4* and, in turn, affect the expression of some of its targets. Further studies are required to investigate the veracity this hypothesis.

Our study provides supporting evidence of a linkage signal to the strong association results of rs613872. This not only further confirms the importance of a locus on chromosome 18 in influencing FECD risk, but also indicates that a causal variant for FECD may be identified within this locus. It is highly possible that rs613872 is tagging a rare variant within this locus that plays a role in the etiology of FECD. Additional sequencing studies using family datasets that are highly linked to this locus is a potential strategy to identify the biological mechanisms underlying how this locus influences FECD pathogenesis.
